# Elicitation of health state utilities associated with varying severities of flares in Systemic Lupus Erythematosus

**DOI:** 10.1186/s12955-015-0262-0

**Published:** 2015-05-28

**Authors:** C. Pollard, S. Hartz, S. Liu Leage, M.A Paget, J. Cook, A. Enstone

**Affiliations:** Adelphi Values, Adelphi Mill, Bollington, Macclesfield, SK10 5JB Cheshire, UK; Eli Lilly, Erl Wood Manor, Windlesham, GU20 6PH Surrey, UK

**Keywords:** Systemic Lupus Erythematosus, Flare, Utility and health state

## Abstract

**Background:**

Systemic Lupus Erythematosus (SLE) is characterised by fluctuating periods of minimal disease activity and ‘flare’. Flare is an important outcome variable impacting the disease burden associated with SLE. The objective of this study was to obtain population-based utility values for varying severities of flare to measure the impact on health-related quality of life (HRQoL) in Australia, Canada, France, Japan, Spain and the UK.

**Methods:**

Six health states (HS) for varying severities of flare were developed based on literature, patient blogs, and interviews with patients (n = 12), rheumatologists (n = 7) and nurses (n = 2). HS were validated by independent clinical experts (n = 6) and pilot interviews (n = 10, UK). HS were evaluated using the time-trade-off (TTO) method during face-to-face interviews with a minimum representative sample (n = 100) of the general population, per-country. Visual Analog Scale (VAS) scores were obtained to validate TTO scores. TTO scores were converted into utility values.

**Results:**

The highest mean TTO utility scores were observed for the anchor HS (minimal disease activity) across all countries; means ranged from 0.66 in Japan to 0.82 in UK. All flare HS were associated with a disutility compared with the anchor HS (p < 0.001), means ranged across countries: mild flare HS: 0.55–0.71, moderate flare HS: 0.38–0.53, severe renal flare HS: 0.33–0.45, severe central nervous system (CNS) flare HS: 0.30–0.45 and severe generalised flare HS: 0.19–0.33. Mean VAS scores followed the same trend.

**Conclusions:**

These results show increasing severity of flare has a detrimental impact on HRQoL. The severe generalised flare HS received the lowest mean utility score suggesting that the perceived day-to-day impact of a severe generalised flare was greater than a severe CNS or severe renal flare. This is, to the best of our knowledge, the first utility study to assess varying severities of flare in SLE across six different countries.

## Background

Systemic Lupus Erythematosus (SLE) is a chronic, multi-system, autoimmune disease which predominantly affects women (>90 %) [1]. SLE is associated with the inflammation and subsequent damage of multiple organ systems, notably the skin, joints, heart, lungs, kidneys and central nervous system (CNS) [1]. The manifestations of SLE are wide-ranging, including but not limited to rashes (predominantly on sun exposed areas), photosensitivity, polyarthritis, ulcers and fatigue [1]. SLE patients typically experience multiple manifestations [1].

SLE is characterised by periods of fluctuating disease activity, a patient can experience periods of minimal disease activity and periods of ‘flare’ [1]. Changes in disease activity are captured using validated indices such as British Isle Lupus Activity Group (BILAG) index [2, 3] and Safety of Estrogens in Lupus Erythematosus National Assessment version of the Systemic Lupus Erythematosus Disease Activity Index (SELENA-SLEDAI) [3, 4]. Severe flares are associated with organ damage accrual and mortality [3]. Management of SLE is increasingly aimed at the minimisation of disease activity or treatment-related adverse events which may contribute to irreversible organ damage and an increase in co-morbidities [5, 6, 7].

Flare has been shown to be an important outcome variable impacting the burden of disease associated with SLE, including patient-reported health-related quality of life (HRQoL) and economic burden. Major organ flares such as those involving the kidneys or CNS are associated with increased healthcare resource use, including hospital admissions and procedures [7, 8]. The clinical burden of severe renal flares is substantial however, previous studies have demonstrated no significant difference in HRQoL between patients with and without renal involvement [9].

Previous studies have shown musculoskeletal flares and subsequently joint pain are a significant predictor of low HRQoL in SLE patients [10, 11]. Patients experiencing high levels of pain were also burdened with greater fatigue, anxiety and depression, than SLE patients experiencing low levels of pain [12]. Fatigue, anxiety and depression are associated with a detrimental impact on patient HRQoL [12–14].

Health utilities play an important role in health economic evaluations and provide a method of assessing the impact of SLE flare on HRQoL. Health utilities are values that define an individual’s preference for a specific health outcome, and are used alongside clinical outcomes to define quality-adjusted life years (QALYs) in health economic evaluations.

Reimbursement bodies recommend indirect elicitation of utilities using the EQ-5D, however when this instrument is deemed insufficient direct valuation techniques can be used [15–18]. There are a number of alternative direct valuation techniques. The Time Trade Off (TTO) method, administered via face-to-face interviews with the general public is the preferred valuation technique by the National Institute for Health and Care Excellence (NICE), when the EQ-5D is not suitable [18].

The objective of this study was to obtain population-based utility values for varying severities of SLE flare to measure the impact on HRQoL for Australia (AU), Canada (CA), France (FR), Japan (JPN), Spain (ES) and the United Kingdom (UK). This is, to the best of our knowledge, the first utility study to assess varying severities of flare in SLE across six different countries.

## Methods

### Study design and rationale

**Fig. 1:**
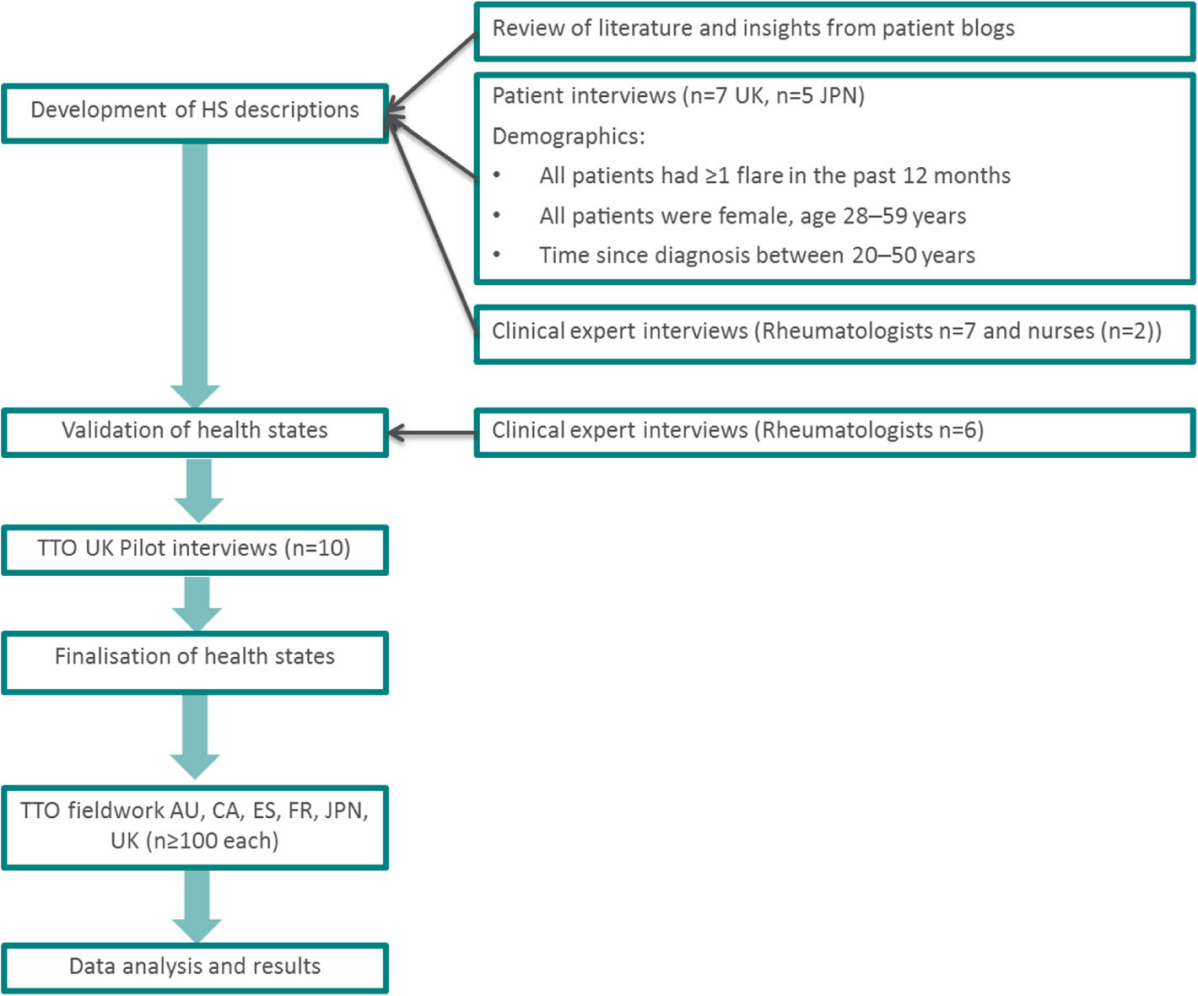
Overview of study methodology

A cross-sectional study, using the TTO method, was conducted with a minimum of 100 members of the general public in AU, CA, ES, FR, JPN and the UK [19]. EQ-5D data collected within randomised controlled trials may not optimally capture the disutility associated with SLE flares [20] when administered at set time points which may not have coincided with a patient experiencing a flare. In addition the EQ-5D may fail to capture the anxiety experienced between flares or adverse events associated with treatment [20]. Therefore direct elicitation was considered an appropriate tool for elicitation of utility values. The TTO method was considered to be the most appropriate direct elicitation technique versus standard gamble (SG). SG is associated with numeracy issues and risk aversion when asking the participant to “bet” on death [21]. Of significance to this study, SG has been shown to be less responsive to musculoskeletal pain [21]. An overview of the overall study methodology is presented in Fig 1.

### Development of health states

Six HS for varying severities of flare were developed based on published literature relating to HRQoL in patients with SLE, patient blogs, interviews with patients (n = 12) and interviews with a group of clinical experts (rheumatologists (n = 7) and nurses (n = 2)).

A recruitment agency was used to recruit patients in the UK (n = 7) and JPN (n = 5); a patient screener was used to determine eligibility for the study. Patients from the UK and JPN were recruited due to potential differences in disease perception and management across countries, as the greatest degree of cultural disparity was expected between these two populations. To ensure accurate recall of experience of symptoms and impacts during a flare, all patients had to have had one or more flares within the past 12 months and have consulted with their doctor regarding the flare(s). Semi-structured telephone interviews with patients provided insights into the symptoms associated with varying severities of flare and the impact of flare on patients’ HRQoL. A selection of quotes from the patient interviews are presented in Table 1. Semi-structured telephone interviews with clinical experts provided additional insight into the symptoms associated with different organ manifestations and varying severities of flare.

The HS aimed to provide a simple and informative description of flare and reflected the experience of a hypothetical SLE patient, aged 41 years (based on mean age of SLE patients in published studies) [1, 3, 6, 22]. Given the heterogeneous nature of SLE, the number of HS needed to represent different organ manifestations (see Table 2) across varying severities of flare and had to be balanced with other factors such as unnecessary complexity and respondent fatigue. To capture increasing severities of flare, six HS were developed including three separate HS for severe flare (severe generalised flare, severe renal flare, severe CNS flare). HS were based on the EQ-5D descriptive system of mobility, self-care, pain, usual activities and emotional elements (see Table 1).

**Table 1:** A sample of patient quotes used to inform the development of the health states and a brief description of the symptoms and impacts included in each health state

Health state	Symptoms	Brief description of impact	Patient quotes
Anchor	Mild joint pain	Mild joint pain, minimal impact on mobility	“It’s frustrating, people don’t think you are ill”
	Sensitive skin	Sensitive skin may impact usual activities as must minimise exposure to the sun	
	Mild fatigue	Fatigue may impact usual activities i.e. change plans with friends	“If I do something [go out, exercise], I know everything will be worse the next day”
		Feeling frustrated	
Mild	Moderate joint pain	Moderate joint pain, unable to walk long distances, some difficulty with dexterity e.g. opening jars	“It takes a while for my joints to get moving, everything takes longer”
	Prominent rash and patchy hair loss	Self-conscious of rash and hair loss	“I can’t walk for more than 45minutes- I get too tired”
	Moderate fatigue	Fatigue may impact usual activities i.e. leave work early	
		Feeling worried	
Moderate	Swollen, tender joints	Severe joint pain, difficulty standing and walking i.e. require aid getting in and out of the shower	“I hate for my family to see me struggle, I feel like a burden”
	Prominent rash and considerable hair loss	Pain when eating	“pain from head to toe, every bone”
		Chest pain disturbs sleep	
	Mouth ulcers	Fatigue may impact on usual activities i.e. take time off work	“Someone has taken your body and replaced it with an old body”
	Chest pain	Frequent blood and urine tests required	
	Severe fatigue	Medication is increased causing weight gain and nausea	
	Hematological	Feeling anxious and depressed	
Severe generalised	Joint pain is all over	Very severe joint pain, impossible to get comfortable	“I am so tired its debilitating, I have to be carried to the bathroom”
	Flaky rash which may scar and considerable hair loss	Eating and swallowing is unbearably painful	“[You feel like] is this ever going to end”
	Mouth ulcers	Finger ulcers are painful and must be dressed regularly by a nurse	
	Finger ulcers	Chest pain makes lying down uncomfortable and disturbs sleep	
	Sharp chest pain and shortness of breath	Severe fatigue impacts usual activities i.e. stay in bed all day, require carrying to the toilet	
	Severe fatigue	Admission to hospital for several days	
	Hematological	Frequent blood and urine tests required	
		Medication is increased causing weight gain and nausea	
		Long-term monitoring for diabetes, heart attack, stroke and fractures	
		Signed-off work for weeks	
		Feeling anxious and depressed	
Severe CNS	Memory loss	Feeling disorientated and confused	“It feels like my brain has gone through a cheese grater, I can’t speak, I can’t concentrate, I get confused”
	Blurred vision	Admission to hospital for several days	“People think you are stupid”
	Seizures	Investigative procedures including neurological tests and MRI scans	
	Plus the symptoms	Medication is increased causing weight gain and nausea	
	outlined for the	Seizures may impact on usual activities i.e. being unable to drive	
	mild HS	Loss of independence	
		Possibility of permanent mental damage	
		Signed-off work for weeks	
		Plus the impacts outlined for the mild HS	
Severe Renal	Swollen legs	Admission to hospital for several days	“I have to work around hospital appointments, work are accommodating- I don’t know how long this will last”
	Severe headache	Require a kidney biopsy	
	Signs of kidney dysfunction	Immunosuppressant’s to help prevent kidney failure	
	Plus the symptoms outlined for the mild HS	Return to hospital monthly for more tests	
		Possibility of kidney failure and dialysis	
		Signed-off work for weeks	
		Plus the impacts outlined for the mild HS	

CNS- Central nervous system, HS-Health state

**Table 2:** Summary of organ manifestations included within each health state

Health state	Organ manifestation					
	Skin	Joints	Hematological	Heart or Lungs	Renal	CNS
Anchor	√	√				
Mild	√	√				
Moderate	√	√	√	√		
Severe generalised	√	√	√	√		
Severe CNS	√	√				√
Severe renal	√	√			√	

CNS- Central nervous system, HS-Health state

The HS descriptions were verified for accuracy, validity and cultural relevance by an independent expert group of Rheumatologists (n = 6).

### Utility elicitation process

Utility values were elicited via face-to-face interviews using the TTO method with members of the general public in each country. Valuation of the HS by the general public are recommended as resource allocation in a publically funded healthcare system should be weighted by the general public’s perception of disease burden [18]. The HS descriptions were piloted with 10 members of the general public to assess comprehensiveness. The outcome of the pilot study resulted in minor wording changes to the HS descriptions.

Based on other population studies, utilities were elictated from a minimum of 100 people from the general population, for each country to achieve a representative cross-section of society [23]. Geographic and demographically representative samples based on general population statistics were recruited, except for Japan where the Tokyo population was deemed to be representative for utility elicitation (see Table 3). Participants were recruited using a population screener, to ensure demographic quotas were met. Demographic data collected in each country included; gender, age, marital status, education, employment status and income. All interviews were audio recorded; participants provided written consent and were given reasonable compensation for their time (equivalent to £30 across the six countries).

**Table 3:** Participant demographics across countries*

Participant characteristics		AU (n = 100)	CA (n = 108)	ES (n = 100)	FR (n = 100)	JPN (n = 101)	UK (n = 110)
Gender	Male	46 (46 %)	53 (49.07 %)	50 (50 %)	50 (50 %)	55 (54.46 %)	56 (50.91 %)
	Female	54 (54 %)	55 (50.93 %)	50 (50 %)	50 (50 %)	46 (45.54 %)	54 (49.10 %)
Age (years)	<18	0 (0 %)	0 (0 %)	0 (0 %)	0 (0 %)	0 (0 %)	0 (0 %)
	18–20	7 (7 %)	13 (12.04 %)	7 (7 %)	10 (10 %)	5 (4.95 %)	10 (9.10 %)
	21–30	27 (27 %)	21 (19.44 %)	15 (15 %)	17 (17 %)	15 (14.85 %)	22 (20 %)
	31–40	23 (23 %)	17 (15.74 %)	15 (15 %)	19 (19 %)	18 (17.82 %)	22 (20 %)
	41–50	16 (16 %)	17 (15.74 %)	27 (27 %)	16 (16 %)	16 (15.84 %)	18 (16.36 %)
	51–60	13 (13 %)	21 (19.44 %)	22 (27 %)	17 (17 %)	15 (14.85 %)	20 (18.18 %)
	61+	14 (14 %)	19 (17.80 %)	14 (14 %)	21 (21 %)	32 (31.68 %)	18 (16.36 %)

AU- Australia, CA- Canada, CNS- Central nervous system, ES- Spain, FR- France, JPN- Japan, UK- United Kingdom

*Figures rounded to 2 decimal places

Two exercises were completed during the interviews. The Visual Analogue Scale (VAS) exercise was completed as a warm-up to familiarise participants with the HS. The VAS is a scale ranging from 0 to 100 where 0 represents death and 100 represents best imaginable health. Each HS was read out in full to the participant by the interviewer, the participant was asked to rate the HS on the scale of 0 to 100. The participant was also asked to rate their own health at that moment in time on the VAS scale. Own health scores could be used to explore any inconsistencies in VAS or utility scores if required as own health can impact the evaluation of HS [18]. The TTO exercise used a horizontal scale representing 1–10 years in ‘full health’. A hypothetical example was used to explain the TTO concept of trading time to live in a preferential state. Each HS was read to the participant; the participant was then asked to state their preference for 10 years in the HS followed by death or 10 years in ‘full health’ followed by death. A ‘flip-flop/ping-pong’ technique was used, where the participant was offered more or less time in ‘full health’ versus 10 years in the HS until a point of indifference was reached. Trading was further refined into months, weeks and days.

### Data analysis

The TTO scores were recorded as number of years, months and days (≤10 years) for each HS. The scores were decimalised and divided by 10 to give a final utility score between 0 and 1 (e.g. utility score for 5 years 6 months = 5.5/10 = 0.55). A minimum utility score of 0 is where a participant rated the HS comparable to death and a maximum utility score of 1 is where the participant was unwilling to trade any time.

A minimum of 40 % of the audio recordings and the documented VAS and TTO scores from each country were quality checked by an independent analyst. In addition a minimum of 20 % of the score sheets were then cross compared against data input tables for each country, used for statistical programming, by an independent analyst. No participants were excluded from the analysis. Given that this study recruited a representative proportion of the general population in each country all responses were included to provide the most comprehensive societal perspective.

All scores were inputted into Microsoft Excel (version 2010), data were exported and analyses were performed using SAS (version 9.3). Descriptive analyses including mean, median, interquartile range, minimum, maximum and standard deviation of the VAS and TTO scores were derived. Paired, two-tailed t-tests were performed at the 5 % level on the VAS scores and TTO utility scores to test for significant differences between all HS combinations. No multiplicity adjustments were conducted.

### Ethics

The study investigators reviewed publically available guidance within each country to determine if ethics approval was required for utility elicitation interviews. The relevant research ethics services in AU, CAN, ES, FR, UK confirmed that ethics approval was not required. Ethics approval was submitted for the study in Japan; approval was granted November 2013.

## Results

### Study population

In total 619 interviews were conducted across six countries, there were no instances of participants refusing to complete the TTO exercise; scores were recorded for all participants. The demographic split of participants across countries by gender and age is presented in Table 3.

### Utility results

The highest mean TTO utility scores were observed for the anchor HS (minimal disease activity); means ranged from 0.66 in JPN to 0.82 in UK. All flare HS were associated with a disutility compared with the anchor HS (p < 0.001) in all six countries: mean mild flare HS ranged from 0.55 in JPN to 0.71 in ES and UK, mean moderate flare HS ranged from 0.38 in JPN to 0.53 in ES, mean severe renal HS ranged from 0.33 in FR to 0.45 in UK, mean severe CNS flare HS ranged from 0.30 in AU to 0.45 in ES and mean severe generalised flare HS ranged from 0.19 in JPN to 0.33 in ES. Within countries a wide range of scores were recorded by participants as indicated by the minimum and maximum TTO utility scores. Descriptive statistics for each HS by country are provided in Table 4.

**Table 4:** TTO utility scores for each health state across countries

		Health state					
Country		Anchor	Mild	Moderate	Severe generalised	Severe CNS	Severe renal
**AU**	Mean	0.75	0.60	0.41	0.23	0.30	0.35
	SD	0.20	0.26	0.30	0.24	0.26	0.27
	Median	0.79	0.60	0.48	0.14	0.25	0.32
	Min–Max	0.06–1.00	0.00–1.00*	0.00–1.00	0.00–0.93	0.00–0.90	0.00–1.00
**CA**	Mean	0.76	0.65	0.42	0.28	0.35	0.37
	SD	0.23	0.27	0.28	0.25	0.26	0.27
	Median	0.80	0.70	0.43	0.19	0.34	0.32
	Min–Max	0.00–1.00*	0.00–1.00*	0.00–1.00*	0.00–1.00*	0.00–1.00*	0.00–1.00*
**ES**	Mean	0.80	0.71	0.53	0.33	0.45	0.43
	SD	0.24	0.28	0.31	0.28	0.29	0.28
	Median	0.90	0.79	0.53	0.30	0.45	0.42
	Min–Max	0.00–1.00	0.00–1.00	0.00–1.00	0.00–0.93	0.00–0.99	0.00–0.99
**FR**	Mean	0.80	0.64	0.46	0.26	0.34	0.33
	SD	0.20	0.28	0.29	0.25	0.27	0.25
	Median	0.80	0.70	0.50	0.20	0.30	0.35
	Min–Max	0.00–1.00*	0.00–1.00	0.00–1.00	0.00–1.00	0.00–1.00	0.00–1.00
**JPN**	Mean	0.66	0.55	0.38	0.19	0.33	0.36
	SD	0.29	0.27	0.28	0.21	0.27	0.27
	Median	0.71	0.56	0.40	0.10	0.30	0.35
	Min–Max	0.00–1.00*	0.00–1.00*	0.00–0.95	0.00–0.95	0.00–0.95	0.00–1.00
**UK**	Mean	0.82	0.71	0.48	0.29	0.36	0.45
	SD	0.18	0.23	0.26	0.25	0.26	0.27
	Median	0.88	0.74	0.50	0.25	0.33	0.44
	Min–Max	0.01–1.00	0.01–1.00	0.00–1.00*	0.00–0.97*	0.00–1.00*	0.00–1.00*

AU- Australia, CA- Canada, CNS- Central nervous system, ES- Spain, FR- France, JPN-Japan, SD-Standard deviation, UK- United Kingdom

*Scores rounded to 2 decimal places, the minimum score recorded was 1 day (0 when rounded)

There was no signal of the data not being normally distributed, the means were close to their corresponding medians (the highest difference is 0.1 for the anchor HS collected in Spain) and all SD were consistent across countries and HS (ranging from 0.18 to 0.30).

The lowest mean utility score recorded across all countries was for the severe generalised HS. Paired t-tests demonstrated a statistically significant difference between the utility scores for the mild, moderate and severe generalised HS (p < 0.001), across all countries, which suggests the perception of increasing severity of these HS is consistent across countries.

The mean scores for severe CNS flare and severe renal flare HS were consistently higher than the severe generalised flare HS. Paired t-tests demonstrated a statistically significant difference between the utility scores for the severe generalised HS and the CNS flare HS or severe renal flare HS (p > 0.005), across all countries. In the UK and Japan no statistically significant difference was observed between the moderate HS and the renal HS (p > 0.1). Therefore the perceived day to day impact of a severe generalised flare is greater than severe CNS or severe renal flare.

The VAS scores were used to validate the TTO scores. The general trend of the data across all countries suggests both the TTO utility and VAS scores decreased from the anchor health state to the severe generalised health state i.e. with increasing flare severity. The mean VAS score for own health was higher than the anchor HS (84.96 versus 62.91); suggesting SLE without flare also has a detrimental impact on HRQoL.

## Discussion

The objective of this study was to elicit six utility values associated with increasing severity of flare. A decrease in utility was observed with increasing severity of flare. These data suggest, based on valuation by the general population, that flares of any severity are seen to have a disutility over the anchor HS (minimal disease activity).

The lowest utility scores were recorded for the severe generalised flare HS, rather than the severe renal flare HS or severe CNS flare HS. Previously studies have reported no significant difference in HRQoL between SLE patients with and without renal involvement [9]. The clinical severity of severe CNS and severe renal flares is substantial; however clinical measures are based on physiological or pathological measurements and may have only little relationship to patients’ feeling of well-being [11, 24, 14]. These findings are consistent with insights from patient experiences gathered during HS development, as the most bothersome symptoms during flare reported by patients (e.g. joints, pain, fatigue) did not always correlate with clinical severity. These findings highlight the importance of including patient perspective/patient-reported outcomes alongside clinical outcomes in the development of new treatments for SLE.

Furthermore, these results are consistent with the existing literature. The severe generalised HS presented in this study included more severe musculoskeletal, fatigue and pain manifestations versus the severe renal flare HS and severe CNS flare HS. Additionally, the severe generalised HS description included more descriptive statements highlighting the emotional impact of these manifestations e.g. depression and anxiety associated with an inability to carry out daily activities and the perception of being a burden to family and friends. Previous reports suggest that SLE patients experiencing high levels of pain were also burdened with greater fatigue, anxiety and depression, and as a result had a significantly lower HRQoL [12, 14]. Patient-reported fatigue has been shown to have a significant impact on multiple aspects of an SLE patients’ life (emotion, cognition, work, activities of daily living, social activities etc.) [13, 14]. Increased treatment and subsequent cumulative organ damage have been shown to be significant predictors of lower HRQoL scores [25], and treatment-related adverse events have been found to have a negative impact on patient HRQoL in approximately 70 % of patients [26]. Increased medication and hospital visits were included within the flare HS descriptors. Further research to quantify the impact of these factors on the general population’s valuation of the HS descriptions is needed.

Within our study the lowest utility scores were often observed in the Japanese cohort, with the exception of the severe renal flare HS and the severe CNS flare HS. Previous studies, not specific to SLE, have shown EQ-5D derived utility scores vary on average by 0.24 between Japan and the UK – this may be due to cultural disparities [27, 28]. Differences observed in utility scores may be reflective of differences in social preference weights between populations. These variations could be due to the general population’s perception of disease severity and impact [29]; descriptors within the HS may have resonated more with the general population in Japan.

The TTO methodology is a standardised tool for HS valuations by Health Technology Assessment (HTA) bodies [18]. The six HS were developed using interviews with physicians and patients as recommended by NICE; furthermore the HS were validated by independent expert rheumatologists from 5 different countries (AU, CA, ES, FR, JPN) [18]. Previously large differences in elicited utility values have been observed for the same HS when using different interviewers, due to methodological inconsistencies in the TTO exercise [30]. The utility scores elicited within this study are consistent across six countries, suggesting methodical limitations were minimal.

It is acknowledged, despite being a widely accepted method, there are some limitations to the TTO technique. Some authors have raised doubts over willingness to trade life for improved health and whether it reveals true preferences [31]. Although not observed within this study, ease of discussing trading off years has been identified as problematic particularly in religious or elderly populations [18, 32]. In addition, this TTO method did not allow for HS to be valued as worse than dead.

The HS may fail to capture all aspects of HRQoL which may influence valuation by the general public [33]. Descriptors within the HS may resonate differently with members of the general public, therefore it is acknowledged there may be a difference in the interpretation of the HS. However, during the development of the HS, pain and fatigue were both cited as the most bothersome by the patients; this was also reflected in some participants’ TTO scores whereby the moderate HS was ranked as more severe than the severe CNS or severe renal HS. The authors observed during the patient interviews that clinical severity may not correlate with patients HRQoL. For example patients with severe underlying disease activity i.e. severe renal flare, may also experience mild skin and joint manifestations in addition to some indicative symptoms of kidney involvement such as headaches and swollen legs which were often cited as less bothersome.

Due to the heterogeneous nature of SLE, not all organ manifestations could be captured, therefore utility values derived from this study may not be applicable to other SLE manifestations of similar overall clinical severity. The HS did not capture the length or frequency of flares, as this is highly variable by patient, and the standard 10 year timeframe for the TTO exercise did not reflect the variation in actual survival between HS. SLE patients have previously reported considerable uncertainty surrounding their condition in particular relating to the timing, severity and future consequences of flare [34]. Uncertainty is closely related to increased anxiety and a negative impact on patient HRQoL [34]. Younger age is a significant predictor of uncertainty and subsequently of a lower HRQoL for SLE patients, as SLE may affect career development, fertility and ability to raise a family; these consequences have not been explored within this study [34]. Furthermore, a large SLE cohort study demonstrated that over the course of one year patients were more likely to experience periods of persistently active disease (PAD) than a flare episode [22]. The reported frequency of PAD compared to flare warrants further investigation into the disutility associated with PAD.

Utility values have been derived from the general population, in line with HTA guidance, rather than from patients with SLE. To ensure a demographic representation of society a 50:50, female to male ratio was used for utility elicitation, therefore gender neutral HS were developed. SLE predominantly affects females, 90:10 female to male ratio, as such health state development was guided by only female SLE patients. Further analyses by gender may elucidate any differences in perception of the HS by male and female members of the general public.

As a representative sample of the general population was recruited, the distribution of participants’ age differs from that of a typical SLE population. Age has previously been found to have an impact on valuation of HS by the general public [18]. Within the utility elicitation participants must imagine they are a 41 year old SLE patient; however, it is acknowledged that participants will draw upon their own experiences and descriptors within the HS may resonate differently with members of the general public. Own health has also been found to have an impact on valuation of HS [18], although in our sample mean VAS scores for own health were higher than the anchor HS in all countries [35]. Age and own health may be a contributing factor for any differences in perception and willingness to trade.

Despite these limitations this study shows that from a societal perspective increasing severity of flare is associated with a decrease in utility scores. The impact of flare on HRQoL, particularly of certain organ manifestations (e.g. musculoskeletal) is consistent with results reported in the literature. These results can be applied in future cost-utility analyses of healthcare interventions which aim to reduce the frequency and or severity of flares in SLE.

## Abbreviations

AEMPS: Agencia Espanola del Medicamento y Productos Sanitarios
AU: Australia
BILAG: British Isle Lupus Assessment Group
CA: Canada
CADTH: Canadian agency for dugs and technologies in health
CNS: Central nervous system
DGFPS: Directorate of pharmaceutical and health products
EphMRA: European market research association
EORTC QLQ-C30: European organization for research and treatment of cancer quality of life Questionnaire
EQ-5D: European quality of life- 5 dimensions
ES: Spain
ESOMAR: European society for opinion and marketing research
FR: France
HAS: Haute Autorité de Santé
HRQoL: Health related quality of life
HTA: Health technology assessment
HUI: Health utility index
JPN: Japan
MCS: Mental component summary
Mod: Moderate flare health state
NICE: National institute for healthcare and clinical excellence
OH: Own health
PBAC: Pharmaceutical benefits advisory committee
PCS: Physical component summary
HRQoL: Quality of life
QWB: Quality of well-being
RCT: Randomised controlled trial
SELENA: Safety of Estrogens in Lupus Erythematosus
SF-36: 36-item Short-form health survey
SG: Severe generalised flare health state
SLE: Systemic Lupus Erythematosus
SLEDAI: Systemic Lupus Erythematosus Disease Activity Index
SLICC/ACR: Systemic Lupus International Collaborative Clinics/ American College of Rheumatology
SMC: Scottish medicines consortium
SR: Severe renal health state
TTO: Time trade off
UK: United Kingdom
VAS: Visual analogue scale
